# The combined effect of *MTHFR* C677T and A1298C polymorphisms on the risk of digestive system cancer among a hypertensive population

**DOI:** 10.1007/s12672-024-00960-y

**Published:** 2024-04-02

**Authors:** Qiangqiang He, Yaping Wei, Hehao Zhu, Qiongyue Liang, Ping Chen, Shuqun Li, Yun Song, Lishun Liu, Binyan Wang, Xiping Xu, Yuhan Dong

**Affiliations:** 1 Shenzhen International Graduate School, Tsinghua University, University Town of Shenzhen, No. 2279, Lishui Road. Nanshan District, Shenzhen, 518055 Guangdong China; 2 Shenzhen Evergreen Medical Institute, Shenzhen, 518057 Guangdong China; 3 College of Public Health, Shanghai University of Medicine and Health Sciences, Shanghai, 201318 China; 4 School of Science, China Pharmaceutical University, Nanjing, 211198 Jiangsu China; 5 State Key Laboratory of Natural Medicines, Research Center of Biostatistics and Computational Pharmacy, China Pharmaceutical University, Nanjing, 211198 Jiangsu China; 6 College of Pharmacy, Jinan University, Guangzhou, 510632 Guangdong China; 7 Inspection and Testing Center, Key Laboratory of Cancer FSMP for State Market Regulation, Shenzhen, 518057 China; 8 Department of Gastrointestinal Surgery/Clinical Nutrition, Capital Medical University Affiliated Beijing Shijitan Hospital, Beijing, 100038 China; 9 Guangdong Key Laboratory of H-Type Hypertension and Stroke Precision Prevention Research and Development Enterprise, Shenzhen, 518057 China; 10 Institute of Biomedicine, Anhui Medical University, Hefei, 230032 Anhui China; 11 National Clinical Research Center for Kidney Disease, State Key Laboratory for Organ Failure Research, Guangdong Provincial Key Laboratory of Renal Failure Research, Guangzhou Regenerative Medicine and Health, Guangdong Laboratory, Division of Nephrology, Nanfang Hospital, Southern Medical University, Guangzhou, 510515 Guangdong China

**Keywords:** *MTHFR* polymorphisms, C677T, A1298C, Digestive system cancer, Case–control study

## Abstract

**Background and purpose:**

The enzyme methylenetetrahydrofolate reductase (*MTHFR*) plays a crucial role in directing folate species towards nucleotide synthesis or DNA methylation. The *MTHFR* polymorphisms C677T and A1298C have been linked to cancer susceptibility, but the evidence supporting this association has been equivocal. To investigate the individual and joint associations between *MTHFR* C677T, A1298C, and digestive system cancer in a Chinese hypertensive population, we conducted a population-based case–control study involving 751 digestive system cancer cases and one-to-one matched controls from the China H-type Hypertension Registry Study (CHHRS).

**Methods:**

We utilized the conditional logistic regression model to evaluate multivariate odds ratios (ORs) and 95% confidence intervals (CIs) of digestive system cancer.

**Results:**

The analysis revealed a significantly lower risk of digestive system cancer in individuals with the CT genotype (adjusted OR: 0.71; 95% CI 0.52, 0.97; P = 0.034) and TT genotype (adjusted OR: 0.57; 95% CI 0.40, 0.82; P = 0.003; P for trend = 0.003) compared to those with the 677CC genotype. Although A1298C did not show a measurable association with digestive system cancer risk, further stratification of 677CT genotype carriers by A1298C homozygotes (AA) and heterozygotes (AC) revealed a distinct trend within these subgroups.

**Conclusion:**

These findings indicate a potential protective effect against digestive system cancer associated with the T allele of *MTHFR* C677T. Moreover, we observed that the presence of different combinations of *MTHFR* polymorphisms may contribute to varying susceptibilities to digestive system cancer.

## Introduction

One-carbon metabolism (OCM) is characterized by two main branches: one consists of reactions involving purine and thymidine synthesis, and the other is responsible for the synthesis of S-adenosylmethionine (SAM) for methylation reactions, including DNA methylation [1], and both pathways are implicated in human carcinogenesis [2–4]. The enzyme 5,10-methylenetetrahydrofolate reductase (*MTHFR*) catalyzes the irreversible conversion of 5,10-methylenetetrahydrofolate to 5- methyltetrahydrofolate (5-MTHF), the primary circulatory form of folate necessary for methionine synthesis [5]. The 5,10-methylenetetrahydrofolate is the folate cofactor for thymidylate synthase [6], thus *MTHFR* resides at a metabolic branch point for it directs the two folate species, either to nucleotide synthesis or to methylation reaction [7].

To date, many mutations in *MTHFR* have been identified; the most studied variants of *MTHFR* are C677T and A1298C. A C to T substitution at nucleotide 677 results in the substitution of a valine codon for alanine, causing an enzyme activity deficiency [5], thus inhibiting the remethylation of homocysteine (Hcy) to methionine, and affecting folate distribution. Many epidemiological studies have demonstrated that this variant results in increased circulating Hcy levels under the condition of impaired folate status [5, 8, 9], as well as clinical implications of cardiovascular disorders [10]. Of note, the prevalence of the *MTHFR* C677T mutation varies in different ethnicities [11], and it has been reported that Chinese populations have a higher rate of the homozygous variant *MTHFR* 677TT (~ 25%) [12]. The second most common mutation in *MTHFR* is A1298C, which changes glutamate into an alanine residue [13]. Studies on the A1298C polymorphism are limited and less documented than those of the C677T allele. It was determined that enzyme activity is 40% lower for this polymorphism in those with the 1298CC genotype (variant homozygotes) than the wildtype 1298AA genotype, but neither the homozygous nor the heterozygous state is associated with significantly higher plasma Hcy levels [13].

For the past decades, the role of OCM and genetic polymorphisms of the enzymes in folate metabolism has attracted much interest in epidemiological research on cancers [14, 15]. The associations between *MTHFR* polymorphisms and cancer risk have been extensively and widely studied [16–18], however, the results have been inconsistent and controversial. It has been reported that the associations for some cancers depend on the ethnicity and folate status of the participants enrolled [19, 20]. Therefore, we conducted a large-scale, case–control study to investigate the associations between these two common variants of *MTHFR* and the incidence of digestive system cancer in a community-based cohort of Chinese adults. Additionally, we aimed to explore the gene–gene interactions and potential effect modifiers within the OCM pathway.

## Methods

### Study population

The methods and major results of the China H-type Hypertension Registry Study (CHHRS, clinical trial registration number: ChiCTR1800017274) have been reported elsewhere [21]. Briefly, the CHHRS was a community-based, prospective, observational, real-world study that was conducted in Lianyungang, Jiangsu Province, and Rongcheng, Shandong Province, China. Started in 2016, the CHHRS was designed to investigate the prevalence of H-type hypertension and related risk factors in China. Eligible persons were aged 18 years or older with essential hypertension, defined as seated, systolic blood pressure (SBP) ≥ 140 mm Hg and/or seated, diastolic blood pressure (DBP) ≥ 90 mm Hg at the screening visit. There were no pre-specified exclusion criteria, except for those who were unable to participate in the follow-up or who were unable to demonstrate informed consent according to the study protocol. The study was approved by the Ethics Committee of the Institute of Biomedicine, Anhui Medical University, Hefei, China.

### Primary outcome and case ascertainment

The primary outcome of interest in this study was incident digestive system cancer over the follow-up period from 2016 to 2018, excluding any previous history of cancer or tumor as assessed at the baseline study. Cancer or tumor diagnoses for all participants were obtained from the China Center for Disease Control at Lianyungang and Rongcheng. This information was cross-verified through the national health insurance system, which was electronically linked to all hospitalization data. Additionally, self-reports provided by participants during the follow-up visits, which occurred every three months as part of the CHHRS, contributed to the cross-verification process. All reported cancer cases were coded according to the International Classification of Diseases, 10th Revision (ICD-10).

### Selection of matched case–control pairs

**Fig. 1:**
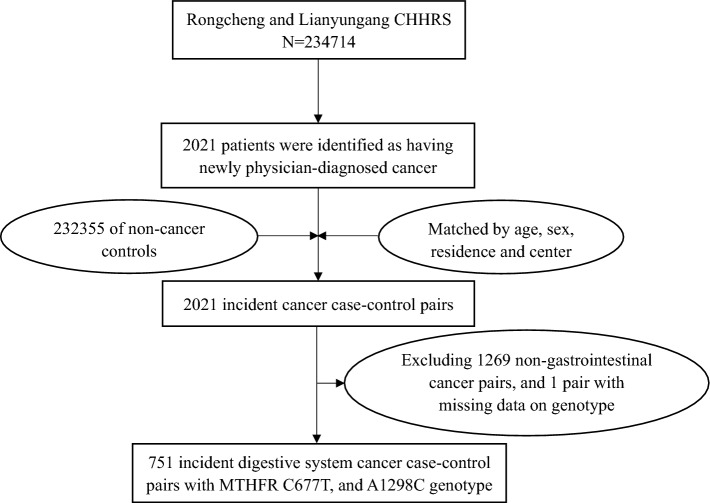
Flow chart of cancer cases and matched controls included in this analysis. CHHRS: China H-type Hypertension Registry Study

To maximize cost efficiency, the present study adopted a nested, case–control design. As shown in Fig. 1, during the follow-up period, 2021 patients were identified as having physician-diagnosed cancer. Controls were selected from the remaining 232,355 participants who were still alive at the end of the study and had never had cancer prior to or during the follow-up period. Controls were matched with incident cancer cases in a 1:1 ratio based on age (± 1 year), sex, study center, and residence at baseline. We excluded 1268 non-gastrointestinal cancer pairs and 1 pair with missing genotype data, resulting in 751 digestive system cancer case–control pairs for the final analysis.

### Exposure assessment

At baseline, all study participants completed a standard questionnaire interview which included information on age, sex, education, occupation, medical history, current medical conditions, and medication intake. Trained research staff measured height, weight, and seated blood pressure according to the standard study protocol. Smoking habits and alcohol consumption status were collected at baseline, and study participants were grouped into three categories: never-smoker/drinker, ex-smoker/drinker, and current smoker/drinker.

### Laboratory assays

Serum Hcy levels (arsenazo-III method) were measured using automatic clinical analyzers (Beckman Coulter); baseline fasting lipids and glucose levels were measured using automatic clinical analyzers (BeckmanCoulter); serum vitamin B12 and total folate levels were measured using a chemiluminescent immunoassay (New Industrial), as published previously [22]. The *MTHFR* C677T (rs1801133) and A1298C (rs1801131) polymorphisms were determined by the Taq Man assay as previously published [22]. Serum concentrations of 5-MTHF were measured by liquid chromatography-tandem mass spectrometry (LC–MS/MS) by the Tailored Medical Laboratory in Shenzhen (Shenzhen, China) following standard lab protocol and vigorous quality control procedures.

### Statistical analysis

Continuous variables were reported as mean and standard deviation, while categorical variables were presented as numbers and percentages. To compare the characteristics of cases and controls, nonparametric Wilcoxon rank sum tests were used for continuous variables, and chi-squared tests were employed for categorical variables. P-values were calculated using a two-sided test. Odds ratios and 95% confidence intervals for the association between *MTHFR* polymorphisms and cancer risk were estimated by conditional logistic regression models. All regression analyses were adjusted for potential confounding variables that may impact the association between the polymorphisms and cancer. This adjustment also considered two specific variables known to be risk factors for cancer, namely smoking and alcohol drinking status.

All statistical analyses were performed using R (version 4.0.5).

## Results

### Study participants and baseline characteristics

As shown in Fig. 1, 751 digestive system cancer cases and matched controls with complete *MTHFR* genotypes data were included in the final analysis. Table 1 presents the baseline characteristics of the participants by case–control status. The mean age of participants was 68.2 years and 65.1% were male. The allele frequencies for *MTHFR* 677-CC, CT, and TT were 21.3%, 57.3%, and 21.4%, respectively among the cases, and 15.3%, 57.5%, and 27.2%, respectively among the controls, the difference between cases and controls was significant (P = 0.002), however, the frequencies of the A1298C (rs1801131) genotypes showed no difference between the two groups. In addition, the proportion of current smoking, former smoking, and never smoking was 32.6%, 11.7%, and 55.7%, respectively for cases, and 26%, 11.7%, and 62.3%, respectively for controls, and this difference was significant (p < 0.014). Other baseline characteristics including education background, Proportion of patients with hypertension, blood pressure, BMI, Fasting glucose, Uric acid, parameters of blood lipid, and serum levels for components of OCM showed no significant differences between the two groups.

**Table 1:** Baseline characteristics of the study participants by case–control status (pairs = 751)

Baseline variables	Total (1502)	Cases (751)	Controls (751)	P Value
Age, year	68.2 ± 11.3	68.2 ± 11.3	68.2 ± 11.3	0.995
Male, No. (%)	978 (65.1)	489 (65.1)	489 (65.1)	1.000
Education, No. (%)				0.912
Primary school or lower	1013 (67.4)	508 (67.6)	505 (67.2)	
Secondary and above	489 (32.6)	243 (32.4)	246 (32.8)	
Smoking status, No. (%)				0.014
Current	440 (29.3)	245 (32.6)	195 (26.0)	
Former	176 (11.7)	88 (11.7)	88 (11.7)	
Never	886 (59.0)	418 (55.7)	468 (62.3)	
Alcohol drinking status, No. (%)				0.685
Current	465 (31.0)	231 (30.8)	234 (31.2)	
Former	90 (6.0)	49 (6.5)	41 (5.5)	
Never	947 (63.0)	471 (62.7)	476 (63.4)	
Hypertension, No. (%)	696 (46.3)	349 (46.5)	347 (46.2)	0.959
*MTHFR* C677T genotype, No. (%)				0.002
CC	275 (18.3)	160 (21.3)	115 (15.3)	
CT	862 (57.4)	430 (57.3)	432 (57.5)	
TT	365 (24.3)	161 (21.4)	204 (27.2)	
*MTHFR* A1298C genotype, No. (%)				0.701
AA	1120 (74.6)	556 (74.0)	564 (75.1)	
AC	353 (23.5)	182 (24.2)	171 (22.8)	
CC	29 (1.9)	13 (1.7)	16 (2.1)	
Clinical measures				
Body mass index, kg/m^2^	25.2 ± 3.6	25.0 ± 3.8	25.3 ± 3.4	0.089
Blood pleasure, mmHg				
Average SBP	144.3 [131.7,158.0]	143.3 [130.0,158.5]	145.3 [133.3,158.0]	0.197
Average DBP	83.0 [75.7,91.7]	82.7 [74.3,92.0]	83.0 [76.0,91.0]	0.391
Laboratory results				
Triglycerides, mmol/L	1.2 [0.8,1.8]	1.1 [0.8,1.8]	1.2 [0.8,1.8]	0.133
HDL cholesterol, mmol/L	1.2 [1.0,1.4]	1.2 [1.0,1.4]	1.2 [1.0,1.3]	0.851
Uric acid, umol/L	311.0 [265.0,366.0]	308.0 [263.0,366.8]	317.0 [268.0,364.0]	0.346
Fasting glucose, mmol/L	5.9 [5.5,6.6]	6.0 [5.4,6.6]	5.9 [5.5,6.6]	0.451
Total cholesterol, mmol/L	6.0 [5.3,7.0]	6.0 [5.2,6.9]	6.1 [5.3,7.0]	0.136
Serum levels for components of OCM				
Hcy, umol/L	12.6 [10.5,15.5]	12.6 [10.3,15.5]	12.7 [10.5,15.4]	0.589
Vitamin B12, pg/mL	516.2 [379.9,680.2]	524.6 [383.8,692.5]	510.1[373.6,661.9]	0.164
Total folate, ng/mL	8.9 [6.1,13.9]	9.1 [6.0,14.0]	8.8 [6.3,13.5]	0.917
5-MTHF, ng/mL	7.9 [5.2,12.7]	8.1 [5.0,13.0]	7.8 [5.4,12.6]	0.805

The summary statistics present N, % for categorical variables, and mean ± standard deviation or median (quartile 1, quartile 3) for continuous variables.

HDL cholesterol, High-density lipoprotein cholesterol; 5-MTHF, 5- methyltetrahydrofolate; SBP, systolic blood pressure; DBP, diastolic blood pressure; Hcy, homocysteine; OCM, one-carbon metabolism

### Association between *MTHFR* C677T, A1298C and the risk of digestive system cancer

For *MTHFR* C677T, as shown in Table 2, after adjusting for potential confounders, compared to participants with the CC genotype, CT carriers conferred a substantially decreased trend for digestive system cancer risk (adjusted OR 0.71; 95% CI 0.52, 0.97, P = 0.034), as did TT carriers (adjusted OR 0.57; 95% CI 0.40, 0.82, P = 0.003; P for trend = 0.003). However, for the variant of A1298C, compared with the AA genotype, the AC and CC genotypes were not significantly associated with changed cancer risk.

**Table 2:** Association between *MTHFR* C677T, A1298C and the risk of digestive system cancer (751 pairs)

*MTHFR* variants	N	Cases/Controls	Crude model		Adjusted model	
			OR (95%CI)	P Value	OR (95%CI)	P Value
C677T genotype						
CC	275	160/115	Ref.		Ref.	
CT	862	430/432	0.71(0.53,0.93)	0.014	0.71(0.52,0.97)	0.034
TT	365	161/204	0.56 (0.41,0.77)	< 0.001	0.57 (0.40,0.82)	0.003
P for trend				< 0.001		0.003
A1298C genotype						
AA	1120	556/564	Ref.		Ref.	
AC	353	182/171	1.08 (0.85,1.37)	0.533	1.05 (0.80,1.38)	0.731
CC	29	13/16	0.82 (0.38,1.75)	0.601	1.17(0.50,2.72)	0.718
P for trend				0.79		0.642

Conditional logistic regression models were used. Adjusted for age, sex, study center, education level, smoking status, drinking status, history of hypertension, SBP, body mass index, pulse, total cholesterol, triglycerides, fasting glucose, Vitamin B12, total folate, and Hcy

CI, confidence interval; OR, odds ratio

### The joint association of *MTHFR* C677T and A1298C with digestive system cancer risk

The joint effects of the two *MTHFR* polymorphisms on digestive system cancer were examined (Table 3). Of note, there were no participants in the subgroup with the 677CT/1298CC combination, nor were there any participants who were homozygous TT at 677 while having a C allele at 1298.

**Table 3:** Number of digestive system cancer cases and controls, *MTHFR* C677T/A1298C allele frequencies, adjusted ORs and 95%CIs for the association of *MTHFR* C677T/A1298C variants with digestive system cancer by *MTHFR* C677T and A1298C with *MTHFR* 677CC/1298AA as reference

*MTHFR variants*		N	Cases/controls	Crude model		Adjusted model	
C677T genotype	A1298C genotype			OR (95% CI)	P Value	OR (95% CI)	P Value
CC	AA	136	78/58	Ref.		Ref.	
CC	AC	110	69/41	1.26 (0.75, 2.10)	0.385	1.01 (0.56, 1.82)	0.983
CC	CC	29	13/16	0.60 (0.26, 1.38)	0.228	0.83 (0.33, 2.10)	0.688
CT	AA	619	317/302	0.77 (0.52, 1.13)	0.185	0.73 (0.47, 1.13)	0.152
CT^a^	AC	243	113/130	0.64 (0.42, 0.98)	0.040	0.64 (0.40, 1.04)	0.070
TT^b^	AA	365	161/204	0.58 (0.39, 0.87)	0.008	0.56 (0.36, 0.89)	0.014

^a^There were no participants with the 677CT/1298CC combination

^b^There were no participants with the 677TT/1298AC or 1298CC combination

Conditional logistic regression models were used. Adjusted for age, sex, study center, education level, smoking status, drinking status history of hypertension, SBP, body mass index, pulse, total cholesterol, triglycerides, fasting glucose, vitamin B12, total folate, and Hcy

**Fig. 2:**
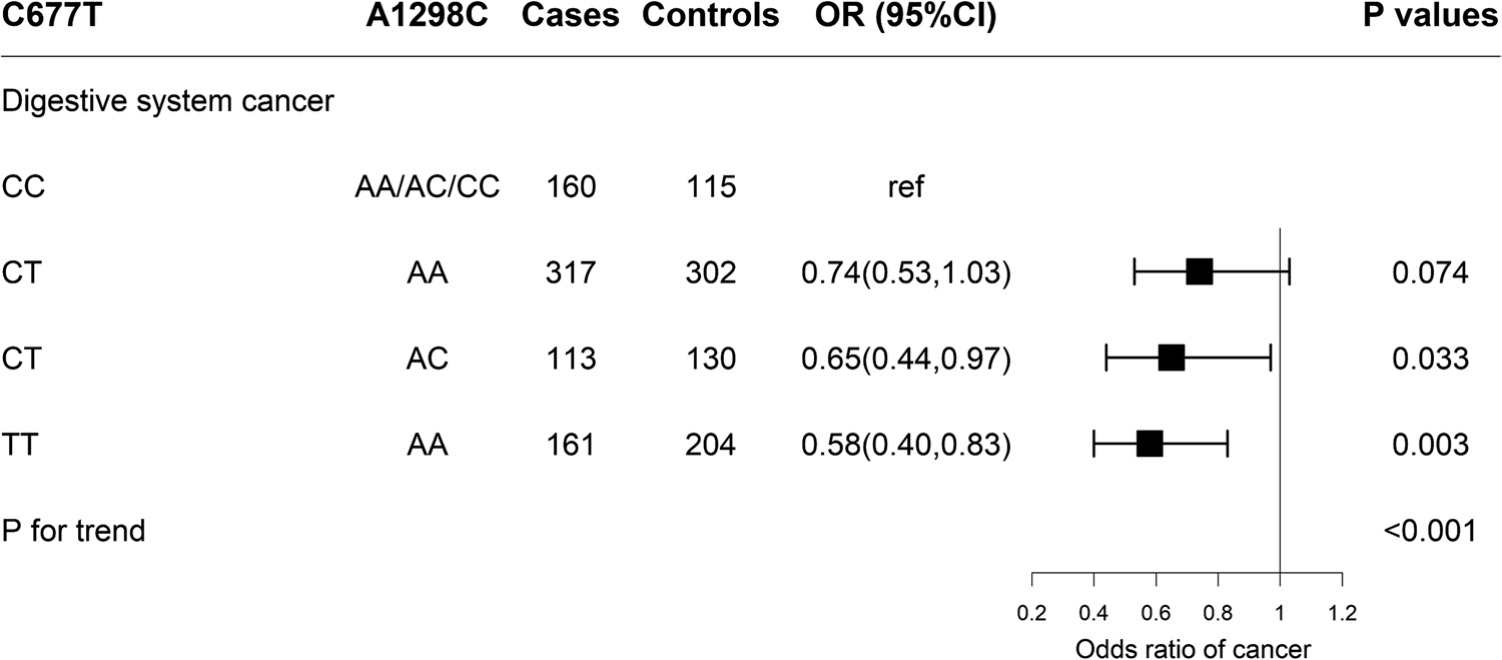
ORs and 95%CI of the combination of the C677T and A1298C polymorphisms on the risk of digestive system cancer. Conditional logistic regression models were used. Adjusted for age, sex, study center, education level, smoking status, drinking status, history of hypertension, SBP, body mass index, pulse, total cholesterol, triglycerides, fasting glucose, Vitamin B12, total folate, and Hcy

Multivariable logistic regression was conducted to evaluate the joint association of C677T and A1298C (Fig. 2). The 677CT genotype accounted for 57.4% of the total population analyzed in the present study. When stratified by A1298C homozygotes (AA) and heterozygotes (AC), a distinct trend was observed where heterozygotes for both the C677T and A1298C mutation, which accounted for approximately 16.2% of case–control pairs, showed a significantly decreased risk (OR = 0.65; 95% CI 0.44, 0.97; P = 0.033), which was similar to that seen among 677TT homozygotes, but the 677CT/1298AA genotypes showed less of a risk reduction (OR = 0.74; 95% CI 0.53, 1.03; P = 0.074).

## Discussion

In the current study, we evaluated the possible associations between the *MTHFR* C677T and A1298C polymorphisms and the susceptibility to digestive system cancer, which is ranked the first prevalent cancer subtype in the CHHRS follow-up period. The T allele of CT677T was found to bring a significantly decreased risk of digestive system cancer among Chinese adults with hypertension.

The relationship between the two *MTHFR* polymorphisms and genetic susceptibility to different types of cancer has been widely evaluated, but the results are controversial. For the C677T variants, and consistent with our results, TT genotype carriers have been shown to exhibit a reduced risk of colorectal cancer [23–25], colorectal adenocarcinomas [26], as well as non-digestive system malignancies, including childhood acute lymphoblastic leukemia [27, 28], oral cancer [29], breast cancer [2], and prostate cancer [30]. In addition, the results of several meta-analysis reviews on colorectal cancer were also consistent with the present study [19, 25, 31]. However, other studies have reported that those with the TT genotype had an increased risk of gastric cancer [32, 33] and colorectal cancer [34], whereas some studies failed to find any association between this variant and the risk of colorectal cancer [35], gastric cancer [36, 37], and other non-digestive system cancers [38, 39].

The conflicting results observed in these studies may be caused by small sample size and inadequate statistical power, or due to demographic differences in the study populations. It has been hypothesized that folate status may modify the relationship between the *MTHFR* C677T polymorphism and cancer. Several case–control studies have shown a more pronounced protective effect for individuals with the 677TT genotype compared with those with the CC genotype in those with adequate dietary folate intake [40], or in populations who live in relatively higher plasma folate regions [20]. This suggests that the impact of *MTHFR* C677T on cancer development is influenced by dietary folate intake. It is possible that the differential ability of folate metabolism between different genotypes of *MTHFR* C677T, as well as variations in the forms of folate present, may not be adequately highlighted due to insufficient substrate availability under conditions of low folate intake. However, high alcohol or low methionine intake has been shown to abolish the reduction in the risk of colorectal cancer in individuals with the TT genotype [23, 41]. Further research is necessary to verify and support the reproducibility of these results, as well as to gain a deeper understanding of their potential underlying mechanisms. The *MTHFR* A1298C polymorphism, however, differed from the C677T, in that we did not observe any significant correlation between this variant and the occurrence of digestive system cancer. This result is in agreement with previously reported results of gastric cancer from meta-analyses [42].

Previous studies have reported that people who are compound heterozygous for the C677T and A1298C alleles, had 50–60% of control enzyme activity, a value that was lower than that of single heterozygotes, and tended to have a biochemical profile similar to C677T homozygous mutation carriers, with lower plasma folate concentrations and elevated Hcy levels [11, 43]. Thus, in our analysis, we explored the combination of the C677T and A1298C polymorphisms on the risk of digestive system cancer for the first time. Interestingly, we found that when the C677T heterozygous genotype was stratified by the A1298C polymorphism, different risks between 677CT/1298AA and 677CT/1298AC genotypes were observed.

The present result is biologically plausible. It has been confirmed that the deficient methylation of dUMP to dTMP and subsequent incorporation of uracil into DNA, could lead to chromosome instability and DNA strand breakage [44], which are often present in human preneoplastic cells and may promote carcinogenesis. The 677TT genotype may reduce cancer risk for it reduces the activity of the thermolabile variant and expands the intracellular pool of 5,10-methylenetetrahydrofolate [45], which is utilized in thymine synthesis, and as a result, the availability of thymine is increased [46] for nucleotide synthesis, and the possibilities of uracil incorporation into DNA are reduced [19, 47]. In agreement with our result, a study examined the combined effect of these two common variants on the *MTHFR* activities, when compared with individuals who have a 677CT/1298AA genotype, significantly decreased activities were observed in individuals heterozygous for both variants (677CT/1298AC) [13]. Therefore, the variability between carriers of the 677CT/1298AA and the 677CT/1298AC genotypes, combined with the comparability between the 677CT/1298AC and the 677TT genotypes may confirm the hypothesis that the cancer risk difference between genotypes stems from disturbances in the folate metabolism pathway, and this disturbances may be driven by the gradual changed *MTHFR* activities.

Despite that the C677T transition has been implicated in many disorders [48–50] and that it might influence the disease profile in some populations, it should not be regarded a priori as a genetic defect that causes disease. While the *MTHFR* 677TT genotype does appear to increase the risk of some diseases, it also appears to protect against others. This may explain the observation showing no correlation between this genotype and longevity [51].

This study has several limitations. Firstly, the generalizability of our findings to other populations with different characteristics may be limited since the study population primarily consisted of rural Chinese individuals with hypertension. Secondly, the specific sample size collected in this study might not have been sufficient to detect subtle and intricate correlations. This highlights the need for larger sample sizes in future studies to validate our results. Furthermore, we failed to confirm the hypothesis that analogous cancer risk of 677CT/1298AC genotype and 677TT genotype participants may be sourced from the similarity of folate metabolism profiles as substantial data missing. In addition, we were unable to analyze detailed cancer subtypes due to sample size limitations, and lack of cytohistologic and histopathological data for further classification.

In conclusion, we observed an inverse association of the *MTHFR* 677TT genotype with digestive system cancer among a Chinese hypertensive population. Our study is the first to demonstrate that different combined genotypes of *MTHFR* polymorphisms may confer significantly different susceptibilities. Considering the high prevalence and lethality of digestive system cancer in China and around the world, these results may guide precise prevention strategies against this fatal disease.

## Data Availability

The datasets generated during and/or analysed during the current study are available from the corresponding author on reasonable request.
